# Comorbid Neurodegeneration in Primary Progressive Aphasia: Clinicopathological Correlations in a Single-Center Study

**DOI:** 10.1155/2022/6075511

**Published:** 2022-09-08

**Authors:** Robert Rusina, Radoslava Bajtosova, Zsolt Cséfalvay, Jiri Keller, Anna Kavkova, Jaromír Kukal, Radoslav Matej

**Affiliations:** ^1^Department of Neurology, Thomayer University Hospital, Prague, Czech Republic; ^2^Third Faculty of Medicine, Charles University, Prague, Czech Republic; ^3^First Faculty of Medicine, Charles University, Prague, Czech Republic; ^4^Department of Communication Disorders, Comenius University, Bratislava, Slovakia; ^5^Department of Radiology, Na Homolce Hospital, Prague, Czech Republic; ^6^Faculty of Nuclear Sciences and Physical Engineering, Czech Technical University, Prague, Czech Republic; ^7^Department of Pathology and Molecular Medicine, Thomayer University Hospital, Prague, Czech Republic

## Abstract

**Introduction:**

Primary progressive aphasia (PPA) is a clinically variable syndrome manifesting as slow progressive loss of speech and language with multiple underlying neurodegenerative pathologies.

**Materials and Methods:**

We included data from nine PPA patients with available autopsies. We then retrospectively reviewed all available medical records, neuropsychology, and MRI results to confirm the corresponding subtypes of PPA and compared them with postmortem neuropathological results.

**Results:**

Clinical presentations corresponded to the nonfluent/agrammatic variant in six cases, the semantic variant in one case, the logopenic variant in one case, and the mixed variant (concomitant nonfluent/agrammatic plus semantic variant) in one case. Patients with a broader clinical presentation, i.e., combining manifestations of one PPA subtype and symptoms of another PPA variant, had autopsy comorbidities showing multiple neurodegenerative disorders. Of the nine subjects enrolled in the study, Alzheimer's disease (AD) was found in eight cases; however, in only one case, AD was detected as an isolated neuropathological substrate of PPA. In eight brain samples, different comorbid neuropathologies were detected: three cases with comorbid AD and dementia with Lewy bodies, two cases with comorbid AD and TDP-43 pathology, one case with comorbid AD and complex tauopathies, and one case with comorbid AD with both tau and TDP-43 deposits. Finally, one case had comorbid tau and TDP-43 pathology but without comorbid AD pathology.

**Conclusions:**

Our observation suggests that PPA cases could be more heterogeneous in their etiology than previously thought and underlying neurodegenerative comorbidities should be considered in routine practice, especially if the clinical presentation of PPA is atypical.

## 1. Introduction

Primary progressive aphasias (PPA) are clinically characterized syndromes with long-term, prominent, and isolated language impairment, progressing over time to dementia. The archetypal PPA subtypes are the nonfluent/agrammatic variant (nfvPPA), the semantic variant (svPPA), and the logopenic variant (lvPPA) [1].

The nfvPPA variant is characterized by agrammatism in language production, effortful speech, and sentence comprehension deficits, with frequently associated parkinsonism and supranuclear gaze palsy. MRI typically shows left fronto-insular atrophy (mainly the inferior frontal gyrus, insula, premotor, and supplementary motor areas) [2]. Anomia and single-word comprehension deficits are the main features of svPPA; MRI atrophy predominates in the left anterior temporal lobe, including the temporal pole [3]. The main symptoms of lvPPA include retrieval (in spontaneous speech and confrontation naming) and sentence repetition skills; MRI sometimes detects atrophy in the left temporoparietal junction area (including the posterior temporal, supramarginal, and angular gyri) [2].

Anatomically, language networks comprise specialized brain areas (network nodes) and interconnected white matter fiber tracts (network connections). The major network of language processing consists of two streams in the dominant hemisphere [4]: the dorsal stream associated with phonological processing (left perisylvian cortical regions, including the inferior frontal gyrus, Rolandic operculum, precentral gyrus, insula, supramarginal gyrus, and superior temporal gyrus and fiber tracts connecting the posterior temporal cortex with the frontal premotor cortex supporting sensory-to-motor mapping and in posterior Broca's area and pars opercularis supporting the processing of complex syntactic structures [5]) and the ventral stream associated with semantic processing (angular gyrus, superior temporal gyrus, and temporal pole, and the intratemporal network-middle and inferior longitudinal fasciculus and the inferior frontal-occipital fasciculus [6]). Dorsal and ventral language network damage may contribute to selective deficits in PPA: nfvPPA with more severe damage to the dorsal stream, svPPA with ventral stream involvement; and in lvPPA; both streams may be partially involved [7].

For clinical diagnosis of PPA subtypes, MRI is helpful in detecting different patterns of focal cortical atrophy. This cortical involvement relates to underlying white matter changes, which can be evaluated by diffusion-weighted imaging. Diffusion tensor imaging, especially tractography, can further assess the changes in the ventral and dorsal stream pathways [7, 8]. Cortical atrophy may be associated with the degeneration of the long fibers, resulting in focal atrophy of the corpus callosum that is pronounced in, for example, globular glial tauopathy (GGT), which is one of the comorbidities found in our cohort [9]. Therefore, in addition to the qualitative data analysis, we used a quantitative approach to tractography and an analysis of the midsagittal area of the corpus callosum.

Other cognitive domains are often involved in PPA: executive dysfunction with impaired set-shifting, verbal fluency, and working memory in nfvPPA [10], visual memory in svPPA (since semantic deficits may impact learning abilities [10], although visuospatial functions remain preserved and svPPA patients may produce very precise drawings [11]), and episodic memory disturbances possibly related to disrupted parietal-hippocampal structural connectivity in lvPPA [12–14]; and visuospatial dysfunction is also affected in lvPPA more than in other PPA subtypes [15].

The underlying neuropathology may differ between PPA subtypes: nfvPPA is often a primary tauopathy (frontotemporal lobar degenerations with tau inclusions (FTLD-tau)), svPPA is a TDP-43 proteinopathy (FTLD-TDP predominantly type C in the harmonized classification) [16], and lvPPA is a focal variant of Alzheimer's disease (AD) [17, 18]. This distribution, however, is not strict: nfvPPA can be due to FTLD-TDP (type A or B) and AD [18–20], svPPA to tau or Alzheimer-type deposits [17, 18], and lvPPA to TDP-43 (type A) or alpha-synuclein deposits (dementia with Lewy bodies (DLB)) [17, 21].

Since PPA subtypes are associated with typical cognitive, MRI, and neuropathological findings, i.e., FTLD-tau with progressive supranuclear palsy (PSP) linked to prominent speech abnormalities with agrammatism and FTLD-TDP type C consistently associated with svPPA [18], specific clinical and MRI features should prompt consideration of an atypical neuropathological diagnosis [20]: DLB has been associated with lvPPA and little to no speech output during the day but with concurrent sleep talking [21] or svPPA with recurrent unprovoked visual hallucinations [22]. Combined neurodegenerative diseases in older age are not uncommon [23]; however, data are more limited in patients with younger onset dementia. Higher comorbid neuropathology for FTLD-TDP type A (50%) than FTLD-TDP type C (12.5%) [24] has been demonstrated. Again, an atypical clinical manifestation increases the probability of comorbid neuropathology, e.g., aphasia with amnesia together with psychosis and parkinsonism was found in a patient with comorbid AD and DLB [25].

Our study aimed to describe PPA cases with available autopsies and correlate language, MRI, and neuropathological data. The interest in this retrospective analysis was driven by neuropathology findings, which in our cases suggest that PPA may be accompanied by comorbid neurodegenerative disease. In clinical practice, in addition to differentiating between the three archetypal PPA subtypes (nfvPPA, svPPA, and lvPPA), efforts should be made to identify possible comorbidities, mainly in cases with atypical presentations. We thus explored the impact of comorbid neurodegeneration on the dorsal and ventral language stream networks, damage to which can considerably influence clinical and neuroimaging presentations.

## 2. Materials and Methods

We retrospectively reviewed data from nine autopsy-confirmed PPA patients: available medical records, speech/language assessment, neuropsychology, and MRI results were compared with neuropathological findings, and the cohort findings were statistically analyzed. The local ethics committee approved the study protocol.

### 2.1. Neurological Examination

All patients were followed in a single center. Data on the first manifestation, neurological status, movement disorders, oculomotor abnormalities, and disease duration were assessed (Table 1).

### 2.2. Language and Cognitive Profile

The evaluation of speech and language processes was carried out by experienced clinicians (neurologists specializing in cognitive neurology and speech and language therapists). The examination did not include standardized aphasia tests or tests of speech apraxia; however, the comprehensive clinical examinations targeted all relevant tasks to capture disruption of any language processes. These language tasks focused on speech fluency, naming objects, single word and sentence comprehension, repetition of words, and syntactically more complex sentences to distinguish between the three archetypal PPA subtypes.

The degree of language deficits is shown in Table 1, where the indication of the presence of a given symptom (“yes”) means marked, i.e., moderate or severe, impairment; very discrete impairments were marked as “mild.” Main aphasia symptoms illustrating the PPA subtype with conclusion are shown in Table 2.

We used a comprehensive neuropsychological battery: for memory, the Repeatable Battery for the Assessment of Neuropsychological Status (RBANS), the Auditory-Verbal Learning Test (AVLT), and the Grober-Buschke test; for executive functions and attention, the Frontal Assessment Battery (FAB), the subtest “similarities” of Dementia Rating Scale 2^nd^ version (DRS-2), the Verbal Design Fluency test (VDFT), the Trail Making Test (TMT), and the Digit Span; and for visuospatial functioning, the Rey-Osterrieth Complex Figure (ROCF), the Clock Drawing Test (CDT), and the copy subtest from RBANS.

Neuropsychological testing was not homogeneous since different neuropsychologists used different neuropsychological tests (from the list mentioned in the previous paragraph). To counterbalance this feature, we first determined *z*-scores for most of the cognitive domains (in screening tests, FAB, or CDT, we used the score from each test) and then calculated standard deviations (SD) for each administered test. Performance relative to deficit was then described as a mild deficit for SD = −1, moderate for SD = −2, and severe for SD = −3.

### 2.3. Neuroimaging

**Figure 1:**
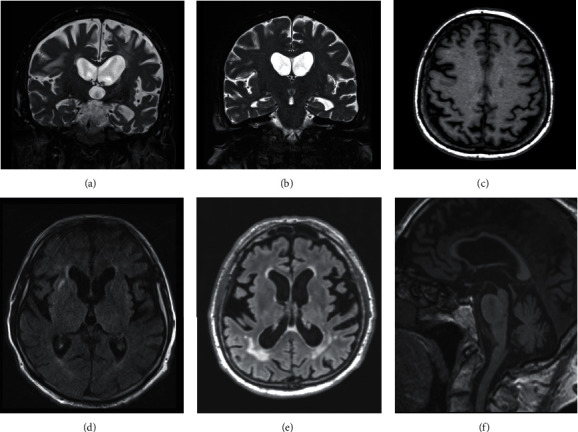
Overview of typical MRI findings in our cases. Asymmetrical atrophy of the opercular and medial temporal lobe was seen in nfvPPA patients ((a) cases 1, 2, 3, and 6; in case 4, temporal atrophy predominated on the right side) and in case 9 with lvPPA. Early left-side anterotemporal atrophy was detected in svPPA patients ((b) cases 7 and 8). In contrast, parietal atrophy of various degrees was rather symmetric in our cases (c), except in case 8. Leukoaraiosis ranged from very mild ((d) Fazekas grade 1) to severe ((e) Fazekas grade 3). GGT comorbidity was associated with atrophy of the mesencephalon (“hummingbird sign”) and with callosal atrophy ((f) cases 3, 6, and 7).

MRI and CT scans were assessed independently by two investigators (J.K. and R.R.) to estimate focal atrophy of different cortical areas and side asymmetry. We used semiquantitative scales to detect focal atrophy in temporal areas (including the hippocampi) and parietal cortices: the mesial temporal atrophy (MTA) scale [26] and the parietal atrophy (PA) score [27]. Cerebrovascular lesions, when observed, were coded using the Fazekas scale [28]. Typical MR findings are displayed in Figure 1.

**Figure 2:**
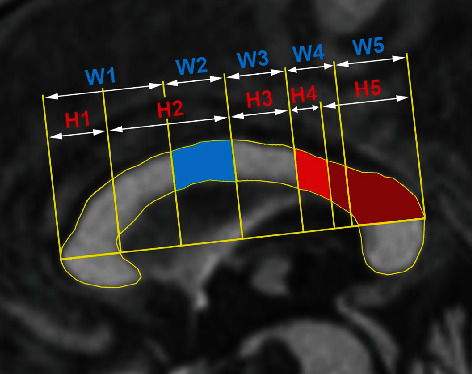
Segmentation of the corpus callosum. Segments of corpus callosum based on Witelson [29] (W1-W5, blue letters) and Hofer and Frahm [30] (H1-H5, red letters) were used for callosal volumetry. The dorsal stream changes are predominantly linked with the W2 segment (in blue), whereas the ventral stream with H4 and H5 (in light and dark red).

Sagittal T1-weighted images (sT1WI) were available for all subjects. In all but two subjects (who had MRI done at another institution with 2D sT1WI), sagittal three-dimensional magnetization-prepared rapid gradient-echo T1 (3D MP-RAGE) sequence was acquired on a single 1.5 MRI (Siemens Avanto, Erlangen) at a high-resolution (1-mm isotropic voxel). We used sT1WI to evaluate the corpus callosum (CC) as a marker underlying tract connection. Quantitative analysis was performed using the ImageJ software to perform manual segmentation of the CC based on two widely used segmentation schemes: Witelson's plus Hofer and Frahm's [29, 30]. After visual inspection, the areas of the respective segments from the callosal bodies were measured in the same plane (Figure 2).

In a subgroup of 5 subjects, DTI data from the same scanner were available. Data were acquired using the standard Siemens echo-planar sequence (2 measurements, each consisting of 1 *b* = 0 and twelve noncollinear diffusion encoding gradient directions at *b* = 1100, TR = 9400 ms, TE = 95 ms, flip angle 90 deg., voxel 2.2 mm isotropic, and 66 slices, PAT 2).

Diffusion-weighted images were processed offline in FSL (FMRIB Software Library [31]). After eddy-current and motion correction, brain extraction was performed using a BET (brain extraction tool [32]); DTIFit was used to calculate the scalar invariants of the tensor and a two-directional probtrackx model for probabilistic tractography [33].

**Figure 3:**
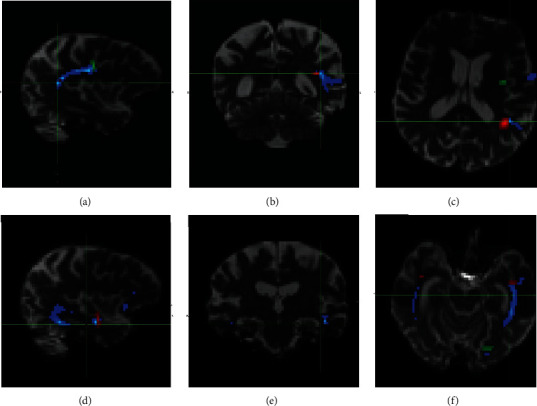
Neuroimaging analysis of the dorsal and ventral streams: probabilistic tractography of superior longitudinal fascicle (SLF, part of the dorsal stream (a–c)) and inferior longitudinal fascicle (ILF, part of the ventral stream (d–f)). Tracts are depicted in blue. In the upper row are the seed masks for SLF in red, and the waypoint mask for SLF is in green; in the lower row, the seed mask for ILF is in green, and the waypoint mask for ILF is in red.

The JHU-White Matter Tractography Atlas (part of FSL) was used to evaluate the structural connectivity of the ventral and dorsal stream: the superior longitudinal fascicle (SLF) and inferior longitudinal fascicle (ILF) were extracted from the atlas and thresholded at a 90% probability. From this thresholded map, seed and waypoint masks were selected in the transverse or coronal plane (the wide mask was applied to the thresholded atlas map), resulting in a pair of seed masks and a pair of waypoint masks (Figure 3). Segmentation was performed in an MNI 2 mm space; the 2-mm brain atlas was then linearly coregistered to the diffusion space of each of the five subjects, and after careful confirmation of the registration result, the transformation matrix was applied to the created masks. ILF and SLF tractography was performed separately for each of the subjects, resulting in a probabilistic map and a waytotal value (a single number corresponding to the total number of generated tracts).

Probabilistic tractography was also performed from the seed cortical masks of regions related to the ventral and dorsal stream, namely Broca's areas BA 44 and BA 45, the inferior frontal gyrus (IFG), insular cortex, the superior temporal gyrus (STG), the precentral gyrus and the supramarginal gyrus for the dorsal stream, and the left angular gyrus for the ventral stream.

As noted in the caption of Table 3, we used the contralateral white matter bundle (corresponding to the less atrophied right-sided nondominant hemisphere) as an internal norm – aware that PPA presents with asymmetric changes in the properties of white matter. We used the ratio of the waytotal of a given tract to the contralateral tract in areas related to the ventral and dorsal streams multiplied by 10^6^ (labeled as PPM in the table). We opted for this approach since comparing the five values to the norms, or a voxel-based approach would have been problematic and less precise. The processing pipeline is (aside from JHU for the generation of masks) a standard FSL approach, similar to a previously published processing algorithm [7]. MRIs were performed close to the same time as the neuropsychological and language assessments (i.e., within one month).

### 2.4. Neuropathology

All patients underwent brain and spinal cord autopsy involving 3–4 weeks of fixation in 10% neutral buffered formalin. Paraffin-embedded tissue sections (4 *μ*m) were examined using routine hematoxylin-eosin and luxol-eosin stains and immunohistochemistry techniques. Following standardized neuropathological procedures for neurodegenerative disorders, samples from both hemispheres, including frontal, parietal, temporal, and occipital cortices, limbic structures (hippocampal region and amygdala), basal ganglia, insula, cerebellum, thalamus, and midbrain, as well as different white matter samples were examined.

**Figure 4:**
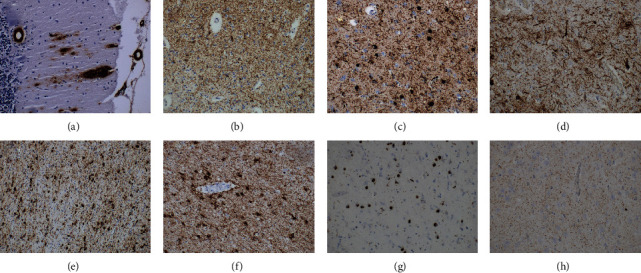
Overview of neuropathological findings in our cases, original magnification 200×. Alzheimer's disease in case 1 ((a and b) similar findings in cases 2–6 and 8–9). Immunohistochemical deposits of amyloid beta-peptide in the form of extracellular plaques of different types and deposits in vessel walls in the form of cerebral amyloid angiopathy in the cerebellum meet the criteria for degree “A3” (a). Immunostaining for hyperphosphorylated tau protein reveals depositions in the occipital cortex–grade VI in the Braak scoring system, which is an example of degree “B3” (b). Prominent Lewy body pathology in case 1 was immunohistochemically positive with antibodies against the pathological form of alpha-synuclein (clone 5G4) in the amygdala ((c) similar findings were seen in cases 2 and 9). Hyperphosphorylated tau protein immunohistochemical depositions (clone AT8) in mesencephalic structures were characteristic of PSP in case 3 ((d) similar findings in case 8). The same immunohistochemical method revealed characteristic globular oligodendroglial inclusions typical for GGT type I in the subcortical white matter in case 7 ((e) similar findings of different degrees in cases 3 and 8). Tau deposits are pathognomonic for ARTAG in the amygdala of case 8 ((f) similar findings in case 3). Hyperphosphorylated TDP-43 protein inclusions in the temporal cortex are characteristic of FTLD-TDP type A in case 5 (g). Hyperphosphorylated TDP-43 protein inclusions in the sclerotic hippocampal region in LATE, case 7 ((h) similar findings in cases 4 and 8).

Immunohistochemistry was performed using antibodies against hyperphosphorylated tau protein (clone AT8, 1 : 500, Thermo Scientific), 4R-tau isoform (RD4, clone 1E1/AG, 1 : 200, Upstate), 3R-tau isoform (RD3, clone 8E6/C11, 1 : 500, Upstate), p62 (clone GP62, 1 : 4000, PROGEN), and ubiquitin (polyclonal, 1 : 500, Dako) with low-pH citrate buffer epitope retrieval, antibodies against amyloid-beta (clone 6F/3D, 1 : 100, Dako), alpha-synuclein (4D6, 1 : 10000, Signet), and phospho-TDP-43 (clone 11/9, 1 : 4000, CosmoBio). Subsequent visualization of all antibodies was performed using horseradish peroxidase–diaminobenzidine (Envision FLEX/HRP, polyclonal rabbit-anti-guinea pig; Dako). Principal neuropathological findings are presented in Figure 4.

AD diagnosis was based on neurofibrillary tangles and amyloid-beta deposits in specific brain regions (using the “ABC” scoring system) [34]. Characteristic neuropathological findings (oligodendroglial and astroglial cytoplasmic inclusions and neuropil threads) were seen in FTLD-tau, namely PSP cases [35], and scored according to Williams et al. [36] and Kovacs et al. [37]. Globular glial tauopathy (GGT), a rare and only recently recognized tauopathy, was diagnosed following the original consensus recommendations [38]. Specific TDP-43 neuronal inclusions and dystrophic neurites were present in specific brain regions in FTLD-TDP cases according to diagnostic criteria for the harmonized classification [16]. A DLB diagnosis used the criteria established by McKeith and Braak based on antibodies against alpha-synuclein [39]. Aged-related diseases were morphologically diagnosed following published diagnostic criteria for aging-related tau astrogliopathy (ARTAG) and limbic-predominant age-related TDP-43 encephalopathy (LATE) [40, 41].

### 2.5. Statistical Methods

The clinical cases were described using 19 clinical variables: MTA scale, PA score, Fazekas scale, memory, attention, executive function, visuospatial as scaled variables, and parkinsonism, oculomotor palsy, nonfluent, semantic, logopenic, poor syntactic complexity, reduced syntactic comprehension, word-finding difficulties, repetition, and speech errors as binary variables (Table 1). Neuropathological findings were studied as ten binary variables based on the final diagnoses, i.e., AD, FLTD, TDP, DLB, PSP, GGT, LATE, ARTAG, and PART (Table 1); as such multiple diagnoses could be separately investigated.

A statistical sample of nine patients was studied using the MATLAB 2019 Statistical Toolbox. The number of cases was very small, and there was no prior knowledge of the statistical distribution; therefore, nonparametric tests had to be used. All clinical variables are binary variables or scores; therefore, parametric tests like the Student's *t*-test and Pearson's *r* could not be applied. Both Spearman rho testing and Fisher's exact contingency table test can be used for small data samples. The resulting critical values were compared using a critical value of 0.05.

## 3. Case Description

### 3.1. Case 1

A 78-year-old man was referred for a two-year history of progressive language disorder, with slowly declining speech fluency and reduced vocabulary (marked lexical retrieval problems). At the formal assessment, very severely impaired language functions were already present (both language production and comprehension). Memory and behavioral manifestation developed subsequently. He started forgetting where he put objects, often repeated meaningless activities, and became apathetic. With disease progression, he became more irritable and neglected personal hygiene. Severe rigidity with impaired vertical gaze and akinetic mutism were late manifestations before death, which occurred eight years after disease onset. nfvPPA was diagnosed. On MRI, brain atrophy was more pronounced on the left side, involving the parietal and especially temporal regions: the medial temporal atrophy (MTA) score was 2, and the posterior atrophy (PA) score was 2, with mild ischemic white matter lesions (Fazekas scale 1). Neuropathologically, this case revealed a combination of both fully developed AD (A3B3C3) and DLB (McKeith II, Braak 5). Vascular changes were focal and relatively prominent.

### 3.2. Case 2

A 68-year-old woman presented with very mild language problems; the beginning of her disease consisted of word-finding difficulties and comprehension difficulties related to complex sentences. Her condition gradually worsened over three years with impaired verbal communication skills and early problems with memory. She had anosognosia and a progressive loss of autonomy. The neuropsychological examination showed reduced verbal production with anomic pauses and articulation disturbance; there was also a significant deficit in memory functions (recognition and recall), a moderate deficit in visuospatial functions, and impaired control of inhibition and shifting. A significant and disproportional worsening of her aphasia was associated with disease progression, as was a loss of insight and comprehension at the sentence level. She also became increasingly anxious and restless. Parkinsonism and slowed oculomotor saccades appeared later; she died eight years after disease onset. She had a family history of dementia (probable AD in her mother and DLB in her brother, however, a genetic investigation for hereditary dementias was negative). We were initially uncertain of the clinical diagnosis but retrospectively concluded nfvPPA. On MRI, pronounced bitemporal atrophy (MTA 3) was prominent in the left hemisphere; parietal atrophy was mild (PA 1), and subcortical vascular lesions were found symmetrically (Fazekas 2). Neuropathologically, a combination of fully developed AD (A3B3C3) with marked cerebral amyloid angiopathy and DLB (McKeith III, Braak 6) was diagnosed.

### 3.3. Case 3

A 76-year-old man developed progressive aphasia, memory difficulties, and fluctuating emotional control. He had diminished verbal expression and impaired repetition of multisyllabic words (speech apraxia), with only monosyllabic vocalization being preserved. He also had severely impaired comprehension skills at the sentence level, but single-word comprehension remained partially preserved. Akinesia with rigidity and supranuclear oculomotor palsy developed progressively, with severe executive dysfunction (stereotypical behaviors, perseveration, apathy, and hyperorality). He died seven years after disease onset. nfvPPA was diagnosed. On MRI, one year after disease onset, marked atrophy was seen in the temporal regions (MTA 3), which was more pronounced on the left side. There was symmetric atrophy of both hippocampi and bilateral parietal atrophy (PA 2); subcortical atrophy was also evident with enlarged lateral ventricles, midbrain atrophy, and white matter lesions that were confluent and diffuse (Fazekas 3). This case was recently published [9]; neuropathologically, there was AD (A2B2C2) and multisystemic FTLD-tau with the predominance of changes related to PSP (Williams score 6-7) and hallmarks of GGT and ARTAG.

### 3.4. Case 4

A 74-year-old woman was referred for slowly progressive language impairment with lexical retrieval in spontaneous speech and comprehension of sentences being primarily impaired. Her written language abilities worsened slowly, and she developed alexia with agraphia). Her autonomy remained largely preserved. Over time, multidomain cognitive deterioration developed, with severe episodic memory deficits, reduced attention, perseverations, stereotypies, loss of emotional control, disinhibition, and agitation. Her verbal communication skills were limited by a severe loss of comprehension. Over the following years, her speech output became increasingly limited. Mild akinesia and gait instability were late findings in the disease course. She died nine years after disease onset. nfvPPA was diagnosed. On MRI, three years after disease onset, we found symmetric atrophy of the parietal regions (PA 2) and temporal atrophy with a mild prominence on the right side (MTA 1) but without vascular lesions (Fazekas 0). Neuropathologically, fully developed AD (A3B3C3) combined with limbic-predominant depositions of TDP-43 protein met the criteria for LATE.

### 3.5. Case 5

A 61-year-old man developed memory and language impairment with a progressive loss of autonomy. Language assessment, after four years of disease progression, revealed severe anomia, reduced syntactic complexity in production with marked agrammatism, impaired sentence comprehension, relatively preserved repetition skills, and speech apraxia. Episodic memory was impaired in both recall and recognition with low learning abilities. With disease progression, partially dopa-responsive parkinsonism with almost symmetric akinesia and rigidity appeared, together with executive dysfunction (apathy, disinhibition, loss of insight and empathy, and perseverative behavior) and mood disturbances (anxiety and depression). He died seven years after disease onset. nfvPPA was diagnosed. On MRI, prominent cortical atrophy was observed in the temporal (MTA 2) and parietal (PA 3) areas, without marked asymmetry and associated with only mild vascular white matter lesions (Fazekas 1). Neuropathologically, a combination of fully developed AD (A3B3C3) with marked cerebral amyloid angiopathy and FTLD-TDP type A (based on the Harmonized Mackenzie classification [16]) was diagnosed.

### 3.6. Case 6

A 74-year-old man who had become depressed and negativistic presented with a history of progressive dysarthria with initially only very mild language involvement (mostly in verbal communication) and memory difficulties. A neurological examination revealed asymmetric predominantly right-sided rigidity and akinesia, with gait instability. The neuropsychological assessment, two years after the onset, showed executive dysfunction, memory issues (recognition and recall), and deficits in visuospatial functions; behavioral manifestations included mainly apathy and depression. The disease progressed into severe dementia with near mutism and severe rigidity. He died about seven years after disease onset. The initial diagnosis was progressive dysarthria, later revised to nfvPPA. MRI showed significant atrophy of the left temporal lobe and left hippocampus (MTA 2), and to a lesser degree, the frontal and parietal (PA 1) areas. Vascular white matter lesions were large and confluent (Fazekas 3). Neuropathologically, a combination of fully developed AD (A3B3C3) with variable vascular changes was diagnosed.

### 3.7. Case 7

A 69-year-old man presented with severely reduced speech production and mild anomia but without language comprehension impairment at the single word and sentence levels. Progressively he developed lexical retrieval problems, speech apraxia, and agrammatism during spontaneous speech. One year later, early memory impairment was present, mainly as delayed verbal recall and total verbal learning ability. Dopa-unresponsive right-sided rigidity, hypokinetic dysarthria, and supranuclear oculomotor palsy with slowed voluntary saccades appeared with disease progression. Oral and written language production and comprehension were impaired at the single word and sentence level; few content words remained in his speech. Later he lost comprehension skills and became almost mute, using only nonverbal communication. He also began to express executive and behavioral symptoms, i.e., perseveration, emotional blurring, alternating apathy and disinhibition, and episodic compulsive laughter. He died six years after disease onset. A combination of two PPA subtypes (i.e., early nfvPPA and svPPA) and hippocampal amnesia was diagnosed. MRI revealed atrophy of both temporal lobes (MTA 3). The left-sided atrophy was associated with pronounced atrophy of the left hippocampus; frontal and parietal regions were relatively spared (PA 1). Moreover, FLAIR hyperintensities, as well as cortical ischemic changes, were present as well (Fazekas 3); midbrain atrophy was also found. This case was previously published [9, 42]; neuropathology revealed full-blown GGT type I combined with age-related neuropathology with deposits of TDP-43 protein, which met the diagnostic criteria for LATE.

### 3.8. Case 8

A 68-year-old woman presented with a very slow onset language disorder lasting over six years; she had marked impairment in speech production (anomia) and especially severe comprehension problems even at the single word level. There was very slow progression to dementia with memory, visuospatial and executive disturbances, apathy, and loss of autonomy. Further behavioral manifestations included social disinhibition, agitation, negativism, and compulsive behavior. Many years later, parkinsonism, together with right-sided spasticity and flexion contractures of the upper extremity, developed. She became mute with only perseverative vocalizations and eventually became completely bedridden. Supranuclear gaze palsy was a late finding. She died at home after an unusually long disease course lasting almost twenty years. svPPA was diagnosed. On MRI, symmetric atrophy of both temporal lobes (MTA 3) was observed. This was associated with parietal atrophy (more on the left side) (PA 3) and unremarkable vascular lesions (Fazekas 0). Neuropathologically, the case revealed fully developed AD (A3B3C3) with a combination of multisystemic FTLD-tau with most changes related to PSP (Williams score 6-7) along with the hallmarks of GGT and ARTAG. Moreover, deposits of TDP-43 protein with a predominantly limbic distribution met the criteria for LATE.

### 3.9. Case 9

A 48-year-old woman presented with progressively worsening speech fluency; she had word-finding difficulties and mild phonemic paraphasia but without comprehension impairment. In parallel, there was gradual memory deterioration and loss of autonomy. Over time she manifested personality changes with irritability, unusual aggressive reactions to familiar stimuli, disinhibition, and compulsivity, together with a lack of insight and empathy. Verbal communication skills became more impaired relative to production and speech comprehension, and her dementia worsened. She died in a nursing home six years after the first symptoms. Her father had also died at the age of 48 with dementia and movement disorders. lvPPA was diagnosed. On MRI, 2–3 years after onset, mild atrophy of the left temporal lobe was the presenting feature (MTA 1). Additionally, there was mild parietal atrophy (PA 1) and minor ischemic white matter lesions (Fazekas 1). This case was previously published [43]; neuropathologically, the case was a combination of two fully evolved neurodegenerations: (1) early-onset AD (A3B3C3) and (2) DLB (McKeith III, Braak 6). Genetic analysis found a presenilin mutation (*PS1* gene).

## 4. Results

Nine PPA subjects (*n* = 9) were included in our analysis. The nfvPPA variant was present in 6 cases (66.67%), svPPA in 1 case (11.11%), lvPPA in 1 case (11.11%), and mixed (nfvPPA+svPPA) in 1 case (11.11%). In two nfvPPA cases, we found atypical associated clinical patterns: one nfvPPA patient initially had progressive dysarthria (case 6), and another developed both early symptoms of nfvPPA and late svPPA (mixed PPA, case 7). The main findings are presented in Table 1.

Parkinsonism (in terms of akinesia and/or rigidity) was found in eight patients (88.89%) and supranuclear gaze palsy in five (55.56%); only one patient manifested spasticity (case 8). All patients with supranuclear oculomotor involvement had neuropathological signs of tau pathology (ARTAG, PSP, or GGT), whereas patients with no proven tauopathy did not develop oculomotor signs.

From our nfvPPA cases with a typical profile of language production impairment and comprehension, four had mild-to-moderate speech apraxia during their disease, and one case experienced spastic dysarthria (based on medical reports and results of speech and language evaluation). This patient, case 6, had marked motor speech problems (dysarthria) from the time of disease onset, and another nfvPPA patient, case 7, developed features typical of svPPA. Two other individuals had fluent speech, one fulfilling the criteria for svPPA, case 8, and the other the criteria for lvPPA, case 9.

Neuropsychological assessments analyzed impairments in the main cognitive domains relative to the value of the standard deviation (severe impairment -3 SD, moderate -2 SD, mild -1 SD). For memory, the mean score was 2.50, and the median was 3.00 [range 1–3, standard deviation (SD) 0.92]. The memory of one subject was unevaluable because of severe impairment of language comprehension. The mean attention score was 2.00, and the median was 3.00 [range 0–3, SD 1.32). For executive functions, the mean was 2.44, and the median was 3.00 [range 1–3, SD 0.88], and for visuospatial functions, the mean was 1.77, and the median was 2.00 [range 0–3, SD 1.20].

Neuroimaging findings included individual focal atrophies that are displayed in the case descriptions, and together with MTA/PA scale scores, objective indices of parietal and temporal atrophy are summarized in Table 1. The mean score was 2.2 (median 2.0, SD 0.83, min 1.0, max 3.0), suggesting the extent of atrophy in the temporal lobe, measured using the MTA scale [24]. The mean score was 1.77 (median 2.0, SD 0.83, min 1.0, and max 3.0), detected using the PA score, which indicated the degree of posterior parietal atrophy [25]. Finally, damage to subcortical white matter was displayed using the Fazekas scale with a mean of 1.55 (median 2.0, SD 1.23, min 0.0, and max 3.0) [26].

Results of CC area measurements are shown in Table 3; the relative numbers are related to normalized CC volumes, with lower numbers suggesting focal white matter atrophy. The dorsal stream corresponds mainly to W2 (and, to a lesser degree, the H3 segments), and the ventral stream is represented by more dorsal parts of the CC, particularly H4 and H5 (Figure 2).

Tractography, available in five cases, demonstrated different structural connectivity of the dorsal stream (represented by the superior longitudinal fascicle) and ventral stream (represented by the inferior longitudinal fascicle) in different PPA subtypes (Table 3, Figure 3). Similar to the CC volume analysis, lower values of the waytotal reflect decreased structural connectivity resulting from a more pronounced impairment of long fiber tracts. Moreover, patients with ARTAG and GGT comorbidity had a lower number of “fibers” in the dorsal stream (a statistical analysis of this phenomenon cannot be performed due to the low number of subjects).

Neuropathology findings revealed an abundance of neurodegenerative comorbidities (Figure 4). AD, the standard background for lvPPA described in the past, was detected as an isolated neuropathological substrate in only one case. All other cases had different comorbid neuropathologies (e.g., tauopathies, synucleinopathies, and TDP-43 proteinopathies). Tables 1 and 2 summarize the associated comorbidities of each patient within the different PPA subtypes. Vascular changes of varying degrees were seen in most cases.

Statistical comparisons of clinical variables and neuropathological findings were also analyzed. Fisher's exact test detected a significant positive relationship between semantic deficits as a clinical variable and ARTAG diagnosis as a neuropathological finding (*p* = 0.0277). The diagonal contingency table contains only two cases where patients had a semantic deficit and an ARTAG diagnosis. The remaining seven cases are patients without semantic deficits and an ARTAG diagnosis without exceptions.

## 5. Discussion

The main findings of our study were as follows: most of the analyzed cases had the nfvPPA variant. All but one had underlying neuropathological comorbidities (AD being the most frequent neuropathological diagnosis), and in some cases, the language profile extended the conventional “pure” description of language impairment typical for the nfvPPA subtype.

All our cases fulfilled the clinical and MRI hallmarks for the respective PPA subtypes [1]. Each PPA variant has been linked to damage to one of the language networks (dominantly dorsal stream involvement in nfvPPA, ventral stream in svPPA, and both, to a lesser extent, with memory impairment in lvPPA). Our findings, however, extended these profiles to include higher levels of complexity, which were probably caused by the comorbid neuropathologies.

In our nfvPPA cases with variable degrees of language deficits, and in concordance with previous findings, we found signs of dorsal tract involvement both clinically (mainly as deficits in syntax and verbal production) and through neuroimaging (reflected as anterior CC atrophy and reduced structural connectivity in the superior longitudinal fascicle, which is the core pathway of the dorsal stream). However, in our nfvPPA cases with postmortem established neurodegenerative comorbidities, we also demonstrated unattended impairment of the ventral stream both clinically (semantic deficits with impaired comprehension) and on neuroimaging (posterior CC atrophy and reduced structural connectivity in the inferior longitudinal fascicle, the core pathway of the ventral stream). In contrast, in the svPPA case, the ventral stream was impaired early on, but the dorsal stream, while affected, was affected to a lesser degree; the lvPPA case had a more proportional involvement of both streams.

Since white matter changes follow gray matter atrophy, we decided to include tractography of SLF and ILF (representing the dorsal and ventral language streams) and an assessment of CC atrophy (as a marker of underlying tract connections). This led us to use CC segmentation (Figure 2) and tractography (Figure 3).

Corpus callosum measurements give insight into interhemispheric connectivity. The quality and quantity of the long fibers provide additional information regarding the impact of focal atrophy on connectivity. Probabilistic tractography was limited by masks derived from the atlas. The distribution of the tracts was, therefore, in alignment with the expected distribution. Tractography values (ppm) were calculated based on the total number of generated tracts not rejected by inclusion/exclusion mask criteria for the impaired and contralateral side for both SLF and ILF. Our interpretation is that decreased ppm values correspond to decreased structural connectivity of the respective tract regardless of the level of global atrophy (which would decrease the waytotal on the corresponding contralateral side).

Currently, little is known about the relationship between neuropathological diagnoses and diffusion properties of the language streams. If present, a pattern of white matter changes associated with PPA could be useful for in vivo diagnosis of specific neuropathological processes. In our study, ventral stream involvement was significantly linked to comorbid ARTAG and GGT neuropathology. However, further research is needed to support this hypothesis. It would be of great value if specific treatments were introduced in the future.

We hypothesize that in patients with a broader clinical presentation corresponding mainly to one of the archetypal PPA variants but also showing symptoms of another PPA variant, neurodegenerative comorbidities should be considered, as demonstrated by case 7, which was a combination of nfvPPA and svPPA attributable to fully developed GGT and LATE which were ultimately confirmed on autopsy [9, 42]. A result of the clinical-neuropathological correlations in our cohort produced a very interesting finding, i.e., that in nfvPPA patients, ventral stream involvement was significantly linked to the ARTAG neuropathology.

Nonlanguage cognitive and behavioral manifestations were evident and progressively worsened into severe dementia during the disease course (Table 1). These manifestations contradict the archetypal concept of PPA as a predominantly language disorder with largely isolated aphasia for 1–2 years combined with slow progression to dementia [44]; however, it is in line with previous reports suggesting early cognitive impairment in PPA patients [10], namely episodic memory disturbance in lvPPA [14]. A possible explanation for our cohort's cognitive and behavioral deterioration was that all but one patient had postmortem confirmed neurodegenerative comorbidities (and the only case with an isolated neurodegenerative pathology–case 6 had AD, which is currently associated with early cognitive and memory impairment [45]).

Our study also focused on PPA motor symptoms. Oculomotor abnormalities were clearly linked to underlying tauopathies (Table 1) [46]. In our cohort, all patients with manifest supranuclear oculomotor palsy had brain tau deposits with variable characteristics (PSP, ARTAG, and GGT), while patients without tau deposits on autopsy showed no oculomotor impairment. Extrapyramidal features (akinesia and/or rigidity) of different degrees were found in all but one patient, irrespective of neuropathology findings.

The main limitation of our study was its retrospective character and heterogeneous clinical and MRI data that made more detailed analyses and correlations difficult. Additional limitations include the small sample size that prevents strong generalizations. Furthermore, since, in most cases, there was no formal aphasia testing, the severity of symptoms was postulated using clinical judgment and post hoc interpretations by speech and language therapists. The single-center design and careful team evaluation, on the other hand, at least partially offset this drawback.

## 6. Conclusions

We found that neurodegenerative comorbidities can affect to different degrees both the dorsal stream (syntax, phonology, typically in nfvPPA) and ventral language stream (semantics, often in svPPA) in the same patient, causing atypical and mixed clinical manifestations. Our observations suggest that in patients that do not fit entirely into the archetypal PPA subtypes, a more concise assessment of the language stream involvement using careful language and MRI analysis (focusing not only on focal cortical atrophy but also using tractography and corpus callosum segmentation) is a useful clinical approach. Underlying neurodegenerative comorbidities should also be considered in routine practice, especially in cases with an atypical clinical PPA presentation.

## Figures and Tables

**(a):** 

Case	PPA subtype	Demographic characteristics			Motor symptoms	
		Gender	Age at onset (years)	Disease duration (years)	Parkinsonism	Supranuclear gaze palsy
1	nfvPPA	M	78	8	Yes	Yes
2	nfvPPA	F	68	8	Yes	Yes
3	nfvPPA	M	76	7	Yes	Yes
4	nfvPPA	F	74	9	Yes	No
5	nfvPPA	M	61	7	Yes	No
6	Progressive dysarthria, later nfvPPA	M	74	7	Yes	No
7	Mixed (nfvPPA+svPPA)	M	69	6	Yes	Yes
8	svPPA	F	68	20	Yes	Yes
9	lvPPA	F	48	6	No	No

**(b):** 

Case	PPA subtype	Language features + speech errors					
		Syntax deficits, agrammatism	Low syntactic comprehension	Anomia	Phonemic errors, paraphasia	Repetition (words, sentences)	Apraxia of speech
1	nfvPPA	Yes	Yes	Yes	Yes	Yes	No
2	nfvPPA	Mild	N/A	Mild	N/A	N/A	N/A
3	nfvPPA	Yes	Yes	Yes	Yes	Yes	Yes
4	nfvPPA	Yes	Yes	Yes	Yes	Yes	No
5	nfvPPA	Yes	Yes	Yes	Yes	No	Yes
6	Progressive dysarthria, later nfvPPA	Yes	Mild impairment	Yes	Yes	Yes	Yes
7	Mixed (nfvPPA+svPPA)	Yes, late mutism	Yes	Yes	Yes	Yes	Yes
8	svPPA	Yes	Yes	Yes	Yes	Yes	Yes
9	lvPPA	Fluent speech	Yes	Yes	Yes	Yes	No

**(c):** 

Case	PPA subtype	Nonlanguage domains				BPSD
		Memory	Attention	Executive function	Visuospatial	
1	nfvPPA	Severe	Severe	Severe	Severe	Apathy, irritability
2	nfvPPA	Severe	Mild	Mild	Moderate	Anxiety, restlessness
3	nfvPPA	Mild	Normal	Mild	Normal	Fluctuating emotional control
4	nfvPPA	Severe	Severe	Severe	Severe	Disinhibition, agitation
5	nfvPPA	Severe	Severe	Severe	Severe	Depression, agitation, anxiety, apathy, disinhibition, irritability
6	Progressive dysarthria, later nfvPPA	Severe	Severe	Severe	Moderate	Depression and negativism
7	Mixed (nfvPPA+svPPA)	Mild	Normal	Moderate	Normal	Apathy, irritability, compulsivity
8	svPPA	N/A	Severe	Severe	Mild	Social disinhibition, negativism, agitation, compulsive behavior
9	lvPPA	Severe	Moderate	Severe	Moderate	Irritability, disinhibition, agitation, compulsivity, negativism

**(d):** 

Case	PPA subtype	MRI				Neuropathology
		Focal atrophy	MTA	PA	Fazekas	
1	nfvPPA	Left temporal, bilateral parietal	2	2	1	AD+DLB
2	nfvPPA	Left temporal, bilateral parietal	3	1	2	AD+DLB
3	nfvPPA	Left temporal, bilateral parietal, midbrain	3	2	3	AD+PSP+GGT+ARTAG
4	nfvPPA	Symmetric parietal,right temporal	1	2	0	AD+LATE
5	nfvPPA	Almost symmetric parietal and temporal	2	3	1	AD+FTLD-TDP type A
6	Progressive dysarthria, later nfvPPA	Left temporal lobe and hippocampus, symmetric parietal, and frontal	2	1	3	AD
7	Mixed (nfvPPA+svPPA)	Left temporal lobe, midbrain	3	1	3	GGT+LATE
8	svPPA	Symmetric temporal, left parietal, midbrain	3	3	0	AD+PSP+GGT+ARTAG+LATE
9	lvPPA	Left temporal lobe	1	1	1	AD+DLB

**Table 2:** Attribution of clinical PPA subtypes based on an evaluation of language alteration and neuropathological summary. Key aphasia features respective to the PPA subtype are italicized for each patient.

Case	PPA subtype	Language impairment characteristics	Neuropathology findings
1	nfvPPA	*Declining speech fluency* and *reduced vocabulary* (marked lexical retrieval problems). Late severely impaired language production and comprehension	Combination of both AD (A3B3C3) and DLB (McKeith II, Braak 5); focal and relatively prominent vascular changes
2	nfvPPA	Initially, *word-finding difficulties* and comprehension difficulties for complex sentences. Progressive *reduction in verbal production with anomic pauses and dysarthria*	Combination of AD (A3B3C3) with marked cerebral amyloid angiopathy and DLB (McKeith III, Braak 6)
3	nfvPPA	*Diminished verbal expression*, impaired repetition of multisyllabic words (*speech apraxia*), with only monosyllabic vocalization preserved. Late severely impaired sentence comprehension, with partially preserved single-word comprehension	Combination of AD (A2B2C2) and multisystemic FTLD-tau with predominant PSP (Williams score 6-7) and hallmarks of GGT and ARTAG
4	nfvPPA	Early *impaired lexical retrieval* in spontaneous speech and sentence comprehension. With disease progression, there was *increasingly limited speech output*	Combination of AD (A3B3C3) with limbic-predominant depositions of TDP-43 (LATE)
5	nfvPPA	*Severe anomia* reduced syntactic complexity in production with marked *agrammatism*, impaired sentence comprehension, relatively preserved repetition skills. *Speech apraxia*	Combination of AD (A3B3C3) with marked cerebral amyloid angiopathy and FTLD-TDP type A (based on the harmonized Mackenzie classification)
6	Progressive dysarthria, later nfvPPA	Progressive *dysarthria* with initially only very mild language involvement (mostly *decreased verbal communication*). Later progressed to severe dementia *near mutism*	AD (A3B3C3) with variable vascular changes
7	Mixed (nfvPPA+svPPA)	*Severely reduced speech production and mild anomia*. Progressively lexical retrieval problems, *speech apraxia*, and *agrammatism* during spontaneous speech. *Late comprehension impairment at the single word and sentence levels. Even later lost comprehension skills and almost mutism*	Combination of GGT type I with deposits of TDP-43 protein consistent with LATE
8	svPPA	Early impairment in production (anomia) and *severe comprehension problems even at the single word level*. Late mutism with only perseverative vocalizations	Combination of AD (A3B3C3) with multisystemic FTLD-tau: predominant PSP (Williams score 6-7), hallmarks of GGT and ARTAG; together with TDP-43 protein deposits consistent with LATE
9	lvPPA	*Worsening speech fluency, word-finding difficulties, mild phonemic paraphasia*; without comprehension impairment. Late impairment in production and speech comprehension	Combination of early-onset AD (A3B3C3) and DLB (McKeith III, Braak 6). Genetic analysis found a presenilin mutation (PS1 gene)

**Table 3:** Imaging correlates of dorsal and ventral stream impairment. Midsagittal area of segments from the corpus callosum, according to two widely used segmentation schemes W: Witelson [29]; H: Hofer and Frahm [30]. Values are in square millimeters as measured on the midsagittal plane. Probabilistic tractography: the waytotal values of superior longitudinal fascicle (SLF) and inferior longitudinal fascicle (ILF) as reported by FSL. SFL and ILF ppm: parts per million, the ratio of the waytotal of the tract to bilateral tracts in the areas related to ventral and dorsal stream multiplied by 10^6^. See the Methods section for the list of ventral and dorsal stream areas.

Case	PPA subtype	Midsagittal area of callosal segments				Probabilistic tractography results				
		W2	H3	H4	H5	SLF	SLF ppm	ILF	ILF ppm	SLF/ILF ratio
1	nfvPPA	51.4	47.9	27.5	123.2	N/A	N/A	N/A	N/A	N/A
2	nfvPPA	80.8	72.7	37.2	164.3	N/A	N/A	N/A	N/A	N/A
3	nfvPPA	35.8	46.5	23.1	140.2	737	1.82	5865	14.48	0.126
4	nfvPPA	74.6	76.1	35.3	143.1	6707	20.87	3382	10.52	1.983
5	nfvPPA	67.2	77.0	37.0	164.5	23756	51.36	19106	41.31	1.243
6	progr. dysarthria, late nfvPPA	43.6	37.7	18.8	95.5	N/A	N/A	N/A	N/A	N/A
7	Mixed nfvPPA+svPPA	40.1	42.2	23.1	107.5	N/A	N/A	N/A	N/A	N/A
8	svPPA	63.9	62.0	29.1	110.9	38	0.10	10167	28.72	0.003
9	lvPPA	81.5	79.2	41.0	127.3	25379	82.80	29328	95.69	0.865

## Data Availability

Data supporting the findings of this study are available on request from the corresponding author.

## Abbreviations

AD: Alzheimer's disease
ARTAG: Aging-related tau astrogliopathy
BPSD: Behavioral and psychological symptoms in dementia
CC: Corpus callosum
DLB: Dementia with Lewy bodies
FTLD-TDP: Frontotemporal lobar degeneration with TDP-43 inclusions
GGT: Globular glial tauopathy
LATE: Limbic-predominant age-related TDP-43 encephalopathy
lvPPA: Primary progressive aphasia-logopenic variant
MTA: Medial temporal atrophy scale (Scheltens)
N/A: Unavailable data
nfvPPA: Primary progressive aphasia-nonfluent/agrammatic variant
PA: Posterior atrophy scale (Koedam)
PSP: Progressive supranuclear palsy
svPPA: Primary progressive aphasia-semantic variant
TDP-43: Transactive response DNA-binding protein of 43 kDa
VaD: Vascular dementia.
